# Abstract of the Proceedings of the Buffalo Medical Association

**Published:** 1862-06

**Authors:** J. F. Miner

**Affiliations:** Secretary


ART. II.—*Abstract of the Proceedings of the Buffalo Medical Association.*

TUESDAY EVENING, April 16, 1862.

Present—Dr. White, President, in the Chair, Drs. Rochester, Congar, Ring, Samo, Harvey, Miner, Jansen, Gay, Butler, Whitbeck, Wyckoff.

The Minutes of the last meeting were read, and approved as published.

*Dr. Rochester* desired to present briefly, for the consideration of the members of the Association, a case which had recently fallen under his care; he more desired to present it, since he understood that cases very similar in character had been observed by others.

April 16, 1862.—Was invited to visit a young lad, 14 years of age, active, vigorous, and healthy constitution. Learned that he had been attending school, had been playing very actively the day before, and while heated considerably, water from a pump had been poured upon his head. When visited at 4 o'clock in the afternoon he was delirious, not knowing what he was doing. Pulse 130 per minute, and very weak. Countenance pale, shrunken and anxious. Learned that he had vomited, and was costive. Messenger came at 7 o'clock, and Dr. Eastman visited the patient for Dr. Rochester, who was sick. Another messenger soon came, desiring Dr. R. to visit the child, also, if possible, as they thought him dying. He died before his arrival.

Previous to death there appeared spots upon the surface like purpura hæmorrhagica. There seemed to be blood change, probably spanæmia.

*Post mortem* examination revealed no satisfactory cause of death. All the organs appeared healthy. Had noticed an account in the *American Medical News*, by Mr. Gull, of Guy's Hospital, of Typhus having again appeared in London more fatal than the Plague. Did not express the opinion that this was Typhus, but had only presented the case for consideration.

*Dr. Miner* would also very briefly report two cases of what appeared in some respects similar disease.

*Case 1st.*—Tuesday morning, March 11th, was invited to visit a servant girl, aged 23 years, living on Niagara street, with Mrs. Playter. The Friday night preceding this visit, she attended a dance, spending most of the night. Saturday she did the washing for the family. Sunday complained of being very tired, but attended church. Monday continued to do the work, but was not well, and passed a restless, sleepless night. Tuesday, when called, she was violently delirious, exceedingly restless, rolling constantly, and wholly unconscious of everything said or done. Pulse could not be counted. Pupils contracted. She seemed to neither see or hear, and paid no attention to what was said to her. Would not open her mouth or take anything. Observed under the skin points of ecchymosis or extravasation of blood; these points were very numerous, observed over the whole surface, and were generally very small in extent, mere points.

*Evening Visit.*—Less delirious; answers questions quite readily and correctly; has taken Dover's powder, prescribed in the morning; speaks hopefully, and thinks she shall be better in the morning; looks very pale and sick.

*Wednesday morning.*—Learned that she slept quietly most of the night; answered rationally when spoken to, and said she would have a cup of coffee. While her attendant was absent for the coffee, she died. The points of ecchymosis had enlarged considerably, and were very distinct, but seemed lighter in color or more yellow. *Post mortem* examination could not be obtained.

*Case 2d.*—March 29th, was called by the Coroner to visit and examine the body of a young lad, son of James Dyer, living on Cedar street. He was ten or twelve years old, naturally very healthy and vigorous. Father and sister testified, "that the day previous to his death he was very sick, and could not sit up. In the after part of the day he talked deliriously, and appeared very strangely; complained of great pain in the head."

The family were moving their residence, and did not pay much attention to the sick boy; "thought he would be better in the morning, and on this account did not call a physician." "He grew worse in the night, vomited several times, and died before morning." They noticed what they called a "breaking out" upon the body, especially upon the neck and arms.

Upon examination of the body after death, nothing unusual was observable, excepting the ecchymosis, which was very distinct, and at once attracted attention, since he had so recently observed it in his other patient who died so suddenly. No *post mortem* examination was ordered by the Coroner, and no jury was called to inform them as to the cause of death.

*Dr. White* had seen three cases; one in his own practice, and two others in consultation; the one in his own, was a type of the others.

Monday morning, Oct. 19th.—Visited John Myer, Oak street, naturally a healthy, robust laborer. Had chill, dyspnœa, quick pulse, great exhaustion. Purpuric spots observed on face, body and limbs; slight hæmorrhage from the gums, great pain in the head, and delirium constant until death. Anodynes, stimulants, tonics and nutriment were freely administered.

Tuesday morning.—Continuance hypocratic, skin sallow, great jactation and slipping down in bed. Died, re-action never having been established. No *post mortem* examination obtainable.

Dr. W. remarked that he was inclined to regard his working in a poorly ventilated grapery, damp and unhealthy, or some malarious atmospheric influence, as the exciting cause of the disease. Had noticed this year a great tendency to a low form of disease, typhoid fever more common than usual, of a low grade, and convalescence slow and tedious; vitality greatly depressed.

*Dr. Ring* had seen two very similar cases to those reported during the last winter, though not so rapid in progress.

*Case 1st.*—A little girl, about eight years old, was taken after dinner with chill, vomiting, and fever. Gave anodynes, and applied cold to the head. Next day was delirious, and very restless. Skin cold and bathed in perspiration. Died at 4 o'clock. Scarlet fever was prevalent, and he called it malignant scarlet fever. Was confirmed in his opinion by his subsequent experience and observation.

*Case 2d.*—A child about the same age and in the same neighborhood, was taken with similar symptoms. An eruption like scarlet fever appeared the second day; it was not very distinct, and soon faded. The termination was the same. Dr. R. remarked that scarlet fever often terminated fatally previous to or without appearance of eruption.

*Dr. Jansen* was called April 17th, in the night, to see a boy who was in great pain in the bowels, which could not be longer endured. Learned that Dr. Haunstein was in attendance, and that he had been called since he lived in the neighborhood. Found on examination, bowels very tender and swollen; legs drawn up, great restlessness, and high fever. Prescribed $\frac{1}{4}$ gr. morphine at this time. At the request of the family, and Dr. Haunstein, was afterwards associate, in the treatment of the case.

The next day the pain was still persistent, notwithstanding they gave $\frac{1}{2}$ grain doses of morphine. The bowels were moved by physic.

On the fifth day of the attack typhoid symptoms were present in marked degree. Stimulants, tonics and food were administered, and the boy is now convalescent.

Wished to inquire if this case could have been typhoid fever from the first? or, was it peritonitis, passing through the stages of the disease, and showing the typhoid symptoms in its progress?

*Dr. Miner* remarked upon the infrequency of typhus or typhoid fever in children, under twelve years of age. This fact was urged as presumptive evidence that the cases of sudden death, reported by Drs. Rochester, White and himself, were not typhus in character, since young lads were the victims in several instances, who he regarded as very unlikely to have such disease, and that he always hesitated before applying the name of typhoid fever to the disease of a child, always fearing that some obscure and unobserved cause was operating to produce the symptoms often termed typhoid fever.

*Dr. White* sympathized somewhat in this opinion, and thought that Dr. Rochester (who was now absent,) did not intend in his remarks to express any opinion of the character of the disease which had proved fatal so suddenly. For himself he certainly did not entertain the opinion that it was malignant typhus, and thought he was sufficiently clear in attributing the death in the case he had reported to depressed condition of the system, induced by hard labor in an unhealthy apartment, &c., &c.

*Dr. Wyckoff* inquired if Dr. Miner designed to say that children never had typhoid fever? and expressed the opinion that cases of that disease were common even with quite young children. Certainly they had symptoms which we could not trace to local cause, perfectly resembling typhoid fever.

*Dr. Miner* replied that typhus and typhoid fever were eminently diseases of adult life, and rarely seen before the period of puberty. In the malignant typhus, called ship-fever, which prevailed a few years since among the emigrants, so extensively and fatally, comparatively few children suffered, though no doubt children exposed directly to the causes of the disease, would in some instances suffer either from it, or some other form of disease, equally fatal. Typhoid fever when prevailing extensively, almost as an epidemic, might include occasionally a child, but many cases which would occur even under these circumstances and be *called* typhoid fever, would under other circumstances, be regarded as some other form of disease. He had rarely seen well marked typhoid fever in a child. He had known them to be sick for a few days, and have fever, furred tongue, headache, &c., &c., but to have typhoid fever in unmistakable type, with the intestinal disease, common, if not essential to it, he had seen it in but very few instances, if at all, and did not believe it of very common occurrence; certainly it was the exception to the general rule.

Dr. Whitbeck was invited to meet with the Society and participate in its transactions.

Dr. T. M. Johnson was proposed for membership.

Voted to adjourn to Tuesday evening, June 3d.

J. F. MINER, Secretary.

